# Congenital Pulmonary Airway Malformation in Preterm Infants: A Case Report and Review of the Literature

**DOI:** 10.3390/life14080990

**Published:** 2024-08-09

**Authors:** Alessia Bertolino, Silvia Bertolo, Paola Lago, Paola Midrio

**Affiliations:** 1 Pediatric Surgery Unit, Cà Foncello Hospital, 31100 Treviso, Italy; alessia.bertolino@studenti.unipd.it; 2 Radiology Department, Cà Foncello Hospital, 31100 Treviso, Italy; silvia.bertolo@aulss2.veneto.it; 3 Neonatal Intensive Care Unit, Cà Foncello Hospital, 31100 Treviso, Italy; paola.lago@aulss2.veneto.it

**Keywords:** congenital lung malformation, CPAM, CCAM, prematurity, very low birth weight

## Abstract

Congenital pulmonary airway malformations (CPAMs) represent a well-known cluster of rare lung malformations affecting 1 in 2500 live births. The natural history of many CPAMs is to increase their size in the second trimester, reach a plateau, and, in about 50% of cases, regress and to become barely detectable during the third trimester. Little is known about cases of affected neonates born prematurely: only six cases are described in the literature, recording different conduct and outcomes. Herein, we report the case of a very low birth weight infant born at GW 28 without antenatal findings and presenting at birth with severe respiratory distress, requiring ventilation. Chest X-rays and a CT scan showed the presence of a solid mass in the left lung. An initial conservative approach was adopted as the baby gained respiratory stability within the first days of life. Routine ultrasound (US) showed a progressive reduction of the lesion, mimicking the process of involution that CPAM can exhibit during late gestation. The rarity of the condition does not allow the formulation of any suggestions regarding one type of management over the other. An initial conservative approach seems to be appropriate with regards to the outcome and possible intra- and post-operative complications.

## 1. Introduction

Congenital pulmonary airway malformations (CPAMs) are a group of rare pulmonary lesions involving the development of the lung during intrauterine life. They appear to affect about 1 in 2500 live births [1] and include cystic adenomatoid malformation (CCAM), bronchopulmonary sequestrations (BPSs), congenital lobar emphysema (CLE), and bronchogenic cysts (BCs).

Among CPAMs, CCAMs are the most common, occurring in 1 in 10,000 live births [2]. They represent a heterogeneous group of cystic or solid, mono, or, rarely, bilobar lung masses. Type 1, characterized by multiple cysts of 2–10 cm or a single large cyst, type 2 with multiple sub-centrimetric cysts, and type 3 with single or multiple cysts < 2 cm, are the most frequent types [3].

The diagnosis of a CPAM during gestation occurs more frequently and earlier than in the past, thanks to the increased use and improved technology of prenatal diagnosis. Approximately 70% of all CPAMs are diagnosed during antenatal screening ultrasound between 18 and 20 gestational weeks [4]. Representative features are the presence of a cystic or solid mass of the lung and, less frequently and later on during pregnancy, polyhydramnios or hydrops due to mass effect. Fetal magnetic resonance imaging (MRI) usually adds little information to an US performed by expert sonographers and gynecologists [5].

At birth, most patients are asymptomatic or pauci-symptomatic, and a long-standing debate on the timing and best approach is present in the literature [6]. If a CCAM, BPS, or BC are confirmed, the infant should undergo surgery within the first year of life. A minority of surgeons suggest a conservative approach in asymptomatic patients [7], but non-operative management is still controversial [1]. In rare cases, newborns are symptomatic [4], and, upon confirmation of diagnosis by means of radiological imaging, they should rapidly be addressed.

Very little is known about the diagnosis and treatment of CPAMs in preterm newborns. Only anecdotal cases have been published, and the management differed among them. As the apparent prenatal resolution or involution of CPAM is a known condition, initial conservative treatment has been proposed [8] as well as prompt intervention if symptoms tend to aggravate [9].

The aim of the present study is to review the literature on the management of CPAM in preterm patients and present one additional case.

## 2. Case Presentation

A baby boy was born at 28 + 4 gestational weeks, weighing 1403 g. The pregnancy had run uneventfully except for an increasing polyhydramnios detected since the 24th week of gestation that reached a maximum vertical pocket of 11 cm. No other abnormalities were noted on US; hence, no second-level diagnostic procedures were required. Spontaneous unstoppable onset of the labor occurred prematurely, so the mother was transferred over, and a complete course of betamethasone for pulmonary maturation and atosiban and magnesium sulfate for neuroprotection, were administered 24 h before delivery. Delayed clamping of the umbilical cord was performed according to current guidelines to reduce a possible adverse neonatal outcome [10].

**Figure 1:**
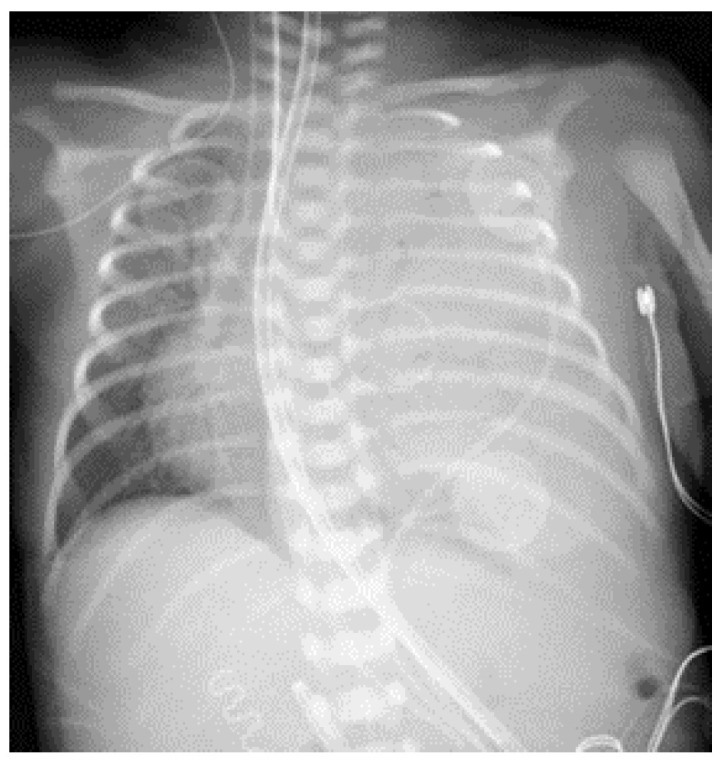
Day 1: chest x-ray showed complete opacification of the left hemithorax with mediastinal shift. Correct localization of the endotracheal tube, naso-gastric tube, and central venous access can be seen.

**Figure 2:**
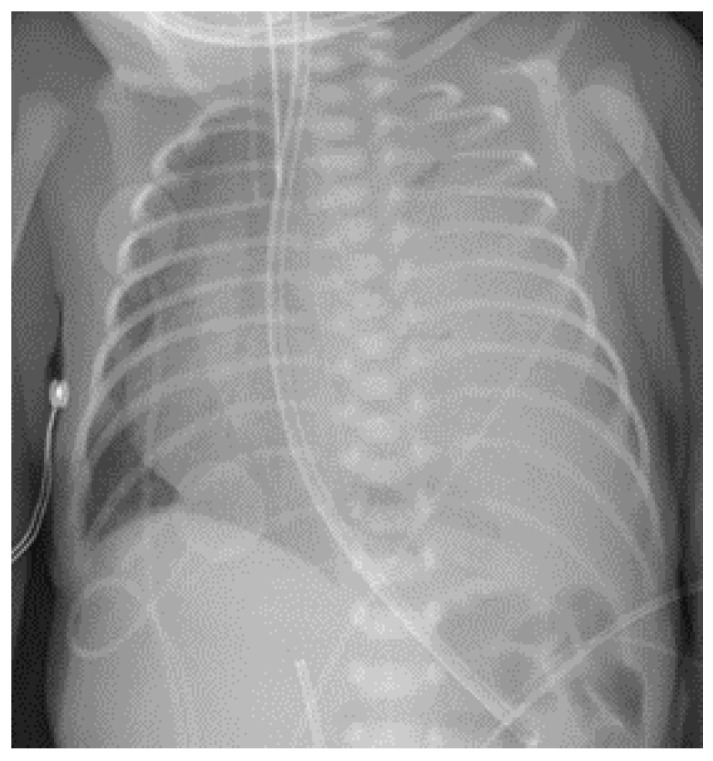
Day 2: after HFOV was applied, no major radiological changes occurred.

Immediately after birth, the patient experienced cyanosis and severe respiratory distress, and Continuous Positive Airway Pressure (CPAP) was started. A facial mask with FiO2 30% was initially applied, followed by intubation and ventilation with positive pressure for progressive respiratory distress. In the Neonatal Intensive Care Unit, he developed severe respiratory distress syndrome, and the ventilator parameters were set at 60% oxygen, tidal volume 6 mL/kg, and peak inspiratory pressure 30 mmHg, that allowed reaching 90% saturation. A chest X-ray (Figure 1) showed almost complete opacification of the left hemithorax and mediastinal shift. Due to worsening respiratory status, FiO2 was increased to 80%, and two doses of endotracheal surfactant were administered, with little effect. The ventilation was shifted to high frequency oscillation ventilation (HFOV), with mild improvement of oxygenation and blood gas parameters (Figure 2). On the second day of life, he developed pulmonary hypertension that was treated with milrinone and opioid sedation.

**Figure 3:**
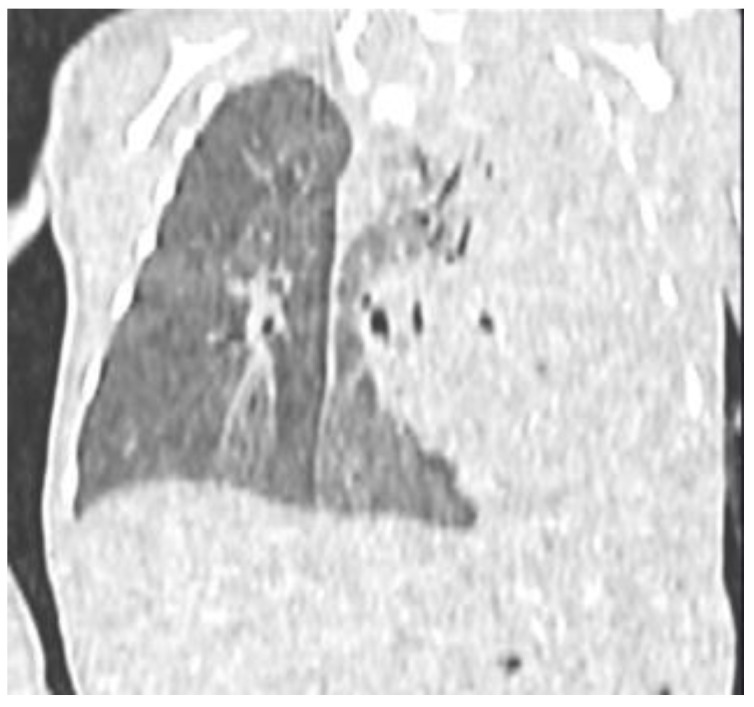
Flash CT scan showing the distribution of the lesion among the majority of the left lung.

**Figure 4:**
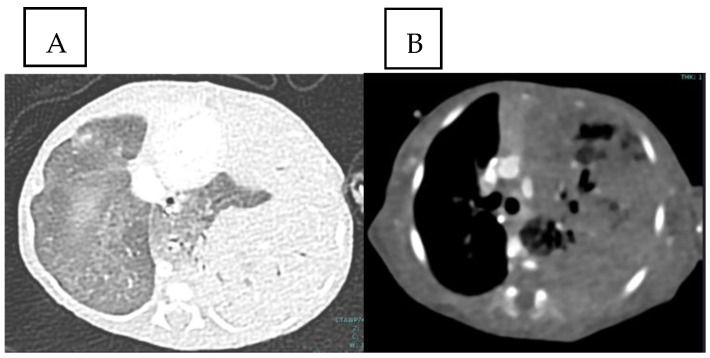
(**A**) Small ground-glass parenchymal opacities were appreciated within the tight lung. (**B**) Solid and cystic components of the mass in the left lung can be seen.

On day 3 of life, a flash CT scan with contrast medium was performed. It showed a hypodense and homogeneous left lung mass, likely belonging to the superior lobe and the lingula, with small areas of spared tissue due to less alveolar and interstitial involvement, an air bronchogram with hypertrophied bronchi, and no abnormal vessels feeding the mass (Figure 3). Small ground-glass parenchymal opacities were observed also within the right lung (Figure 4). The left inferior lobe result was partially spared and antero-inferiorly compressed by the lesion, and the mediastinum was shifted. Based on this imaging, it was not possible to distinguish between a CPAM and a pleuropulmonary blastoma. An initial surgical approach was considered. However, the patient over the next few days remained stable without requiring any further increase of the respiratory parameters. Therefore, the surgeons and neonatologists decided on a wait-and-see approach with strict bedside US follow-up. On US, the following features were recognizable: normal sliding, thickening of pleural lines, confluent B-lines, and hyperechogenic areas of the left lobe. No pleural effusion was detected.

**Figure 5:**
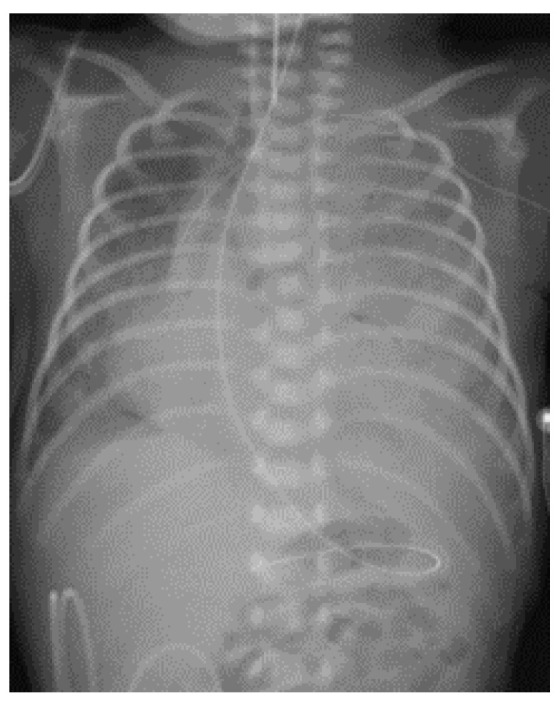
Day 7: improvement of mediastinal shift and lung expansion with reduction of the opacification mainly in the superior hemithorax.

On the 7th day of life, the general conditions of the patient started to improve, and it was possible to shift him onto conventional mechanical ventilation. On the chest X-ray, the superior lobe was slightly more ventilated compared to the previous one (Figure 5). The patient was therefore extubated on day 16 of life and subsequently supported by non-Invasive Mechanical Ventilation (nIMV) for the following month. Eventually, nCPAP was used and, finally, low-flow oxygen.

On day 78 of life, US showed a lung mass measuring 1 × 1 cm with a cranial, small, anechogenic area, suggestive of cystic evolution (0.4 × 0.4 cm). The patient was therefore discharged home on oxygen support by nasal cannula at 0.1 L/min.

**Figure 6:**
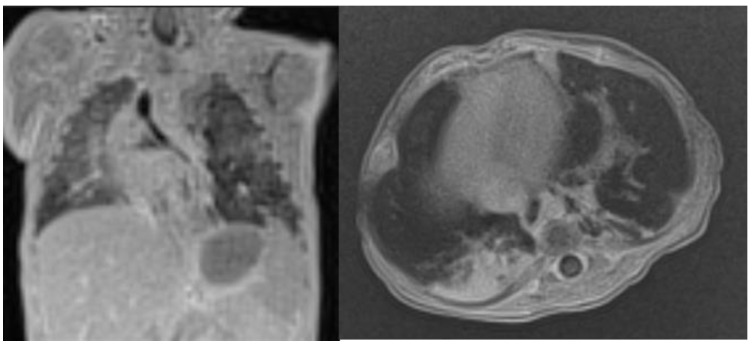
Lung MRI at 3 months of age: the mass of the left lung is barely detectable, and a slight mediastinal shift is still present.

Taking advantage of the expertise in our Centre, at three months of age, an MRI was performed with contrast medium. The left lung appeared much more ventilated due to drastic reduction of the described hypodense mass, and some parenchymal consolidations with blurred, irregular margins and bronchi were still recognizable at the apical and basal segments of the left lower lobe and within the lingula. Finally, another dense area was visible in the paramediastinal region of the left upper lobe (Figure 6).

The patient is now 9 months old and thriving without neurological sequelae or oxygen supplementation. An MRI is scheduled at 1 year of life, and surgical excision is eventually planned based on the imaging findings.

## 3. Discussion

The natural history of CCAM, BPS, and CLE is to increase in size between 20 and 28 weeks of gestation. In particular, CCAM stems from disorders during the pseudoglandular stage of lung development, when the primary bronchial buds undergo a programmed branching to define the tree-like respiratory tract. During the third trimester, the CPAMs tend to plateau and, finally, either to stabilize or progressively apparently disappear later in pregnancy [11].

The CPAM volume ratio (CVR), calculated as the ratio between the volume of the malformation and the fetal head’s circumference, is one of the most popular and valid tools to predict the trend of development of a CPAM. The value of the CVR correlates strongly with the postnatal outcome, and the values have been proved to be reliable for stratifying fetuses into categories of high and low risk of hydrops development and subsequent mortality [12].

Treatment of CPAM during pregnancy is rare. It depends on symptoms and is still controversial [13]. Thoracoamniotic shunts can be considered in cases of large cysts that rapidly increase their volume and compress the mediastinum. In cases of growing microcystic CPAM, cysts that remain large, and onset of hydrops, the administration of maternal steroids may constrain the excessive expansion of the malformation [14] and contribute to lung maturation. Open lobectomy in utero is performed only in a few selected centers in the world because of high fetal and maternal morbidity [15]. Recently, other techniques, such as resection at delivery via an Ex-Utero Intrapartum Treatment (EXIT) or immediately post cesarean section have been showed to produce similar fetal and neonatal outcomes to the the open technique with reduced maternal morbidity [16,17].

In cases of “vanishing CPAM,” the progressive reduction of echogenicity [18] can be documented with US until apparent complete normalization [19]. This phenomenon generally occurs in cases of microcystic lesions and low CVR. The complete regression of a CPAM after birth is rare [20] and could be related to temporary bronchial obstruction by a mucous plug that eventually resolves [21]. These patients need to be studied in the first months of life with CT or MRI to confirm the resolution or presence of CPAM [22].

The majority of patients with a prenatally diagnosed CPAM are asymptomatic at birth and can be discharged without further perinatal examinations, as standard chest X-ray may be falsely normal [1]. However, a follow-up program based on CT or MRI needs to be set to define the characteristics of the CPAM and to plan either a conservative or an invasive approach.

In the minority of patients who present with increasing respiratory distress at birth, a prompt radiological assessment and surgical approach is mandatory. In these cases, an open lobectomy is generally preferred over the thoracoscopic approach.

The risk of malignant degeneration in CPAM is rare, but evidence in the literature suggests a possible association between CPAM and lung cancer in 1–3% of cases [23]. This includes pleuropulmonary blastoma (PPB), bronchioalveolar carcinoma (BAC), and rhabdomyosarcoma (RMS).

Type 1 CCAM is considered the most common CPAM involved in neoplastic transformation, as mucogenic cells populate the cysts and the small bronchiolar-like structures around the cysts [24]. These clusters of mucinous cells have been demonstrated to be at risk of malignant progression to BAC because they contain the oncogenic KRAS mutations in about 30% of cases.

Very little is known about CPAM in preterm babies. Most cases present as isolated malformation [3], and only a minority is affected by other congenital anomalies [25]. Therefore, except for the above-mentioned polyhydramnios and fetal hydrops, the pregnancy is usually carried to term uneventfully.

Nonetheless, to the best of our knowledge, only six cases of CPAM in preterm babies have been described in the literature. A search was conducted in PubMed, Scopus, Scie (Web of Science), and Google Scholar by one of the authors (AB), querying for congenital pulmonary airway malformations (CLMs), CCAM, congenital lung malformations, lung malformations, and preterm from 1 January 1990 to 31 March 2024. Seven articles were retrieved, but one was excluded because the onset of symptoms and surgical management occurred beyond the first month of life (Table 1).

The first paper [26] describes a preterm baby born at 29 gestational weeks without antenatal findings who needed immediate intubation. The chest X-ray showed a dense mass occupying the left hemithorax and mediastinal shift. After initial conservative treatment, respiratory insufficiency occurred, and the patient underwent urgent left thoracotomy and pneumonectomy on day 4 of life. Histological analysis confirmed a type 3 CCAM.

Case 2 [27] refers to an extremely premature baby (23 + 4 GW) without antenatal findings and with twin-to-twin transfusion syndrome. He presented at birth with severe respiratory distress and a large right lower lobe mass on X-ray. After initial successful stabilization and cardiac surgery (for patent ductus arteriosus), at six weeks of life, a CT scan confirmed a cystic lesion, which was surgically excised. The histological specimen showed a persistent pulmonary interstitial emphysema (PPIE). This is not a typical type of CPAM, but imaging, management and surgery did not differ from the typical forms.

Case 3 [28] is a 28 GW baby with a prenatal diagnosis of CPAM, presenting at birth with respiratory symptoms. Due to rapid deterioration, a CT scan was obtained that showed a mass in the right lower lobe with mediastinal deviation. At 9 days of age, a wedge resection was performed, and histology confirmed a CCAM type 1. The patient required further surgery because of post-operative pneumothorax.

In case 4 [8], Abusalah et al. describe a potential CPAM in a preterm twin neonate. No antenatal findings were reported, and delivery by cesarean section at 28 weeks occurred because of intrauterine growth restriction in both twins. At birth, the baby presented with respiratory symptoms and a cyst in the right lower lobe on the initial chest X-ray. The patient was conservatively treated in spite of the enlargement of the cyst, which eventually was no longer detectable at 4 weeks of age.

Case 5 [29] reports a rare case of bilateral CCAM in an extremely preterm patient. The US antenatal findings showed enlarged bilateral lungs from the 20th gestational week. An MRI was also performed at 24 weeks that confirmed the marked lung enlargement and massive polyhydramnios. The patient was delivered spontaneously at 26 + 1 weeks and presented at birth with apnea and bradycardia. Initial conservative treatment was undertaken; despite HFOV, administration of surfactant, and inhaled nitric oxygen, the respiratory status worsened, and the patient died within the first day of life. The post-mortem findings were suggestive of a bilateral type 3 CCAM.

The sixth case [9], similar to case 1, refers to a baby born prematurely at 28 gestational weeks for polyhydramnios present since week 21. A single dose of betamethasone had been administered prior to delivery. The patient presented at birth with bradycardia, progressive severe respiratory distress, and complete opacification of the right lung on X-ray. Initial monitoring by US and a CT scan confirmed the presence of a mass involving the majority of the right lung. Due to worsening ventilatory parameters and increasing inotropic support, surgery was undertaken on day 12 of life. A right upper lobectomy was accomplished through thoracotomy that led to a dramatic respiratory status improvement and, finally, discharge home at 3 months of age on oxygen supplementation. Histology examination confirmed a type 3 CCAM.

Our patient resembles the sixth case in terms of a lack of prenatal diagnosis, prenatal care, initial respiratory status, and thoracic imaging. In a difference, he received a complete course of betamethasone prior to delivery that may explain the spontaneous improvement over the first days of life instead of the gradual deterioration and surgical approach required in the previous case. Moreover, the present case differs from all the others as it is the only one in which successful conservative management was adopted, in spite of the persistence of the lesion. The possibility of postponing the surgery in these fragile patients should always be pursued in order to reduce complications and improve the outcome. The options that are available during pregnancy, such as the insertion of a thoracic drain in cases of cystic lesions or the administration of steroids for rapid growing solid masses, could be successfully adopted also after birth. Indeed, our case, born at the time of the maximum expansion of the mass, benefitted from a complete course of steroids that would have likely been administered to the mother if the pregnancy had continued.

The cases described suggest that the natural history of CPAM during prenatal life can be observed also in postnatal life in preterm babies, mimicking the trend to plateau and reduction of the mass observed in many fetuses with CPAM during the third trimester. However, the respiratory symptoms of premature patients affected by CPAM should be anticipated to be more severe compared to term babies with CPAM because of lung immaturity.

## 4. Conclusions

In conclusion, although little data are available, preterm babies presenting at birth with unexpected respiratory symptoms should be quickly addressed using X-ray, US, and, eventually, more advanced radiological procedures. If a CPAM is suspected, the fact that up to 15% of solid CPAMs can stop growing and regress later in gestation should encourage an initial conservative approach to allow for neonatal growth and improvement of respiratory and general status. In these rare cases, surgery can be delayed upon radiological confirmation of CPAM to an age and weight that enable the patient to undergo the operation safely.

## Figures and Tables

**Table 1:** Crucial information from the articles retrieved in Literature and our current study.

	Referral	Prenatal Findings	GW at Birth	Respiratory Symptoms at Birth	Surgical Neonatal Intervention	Type of Surgery/Histology	Outcome
Case 1	Prendergast et al., 1998 [26]	No	29	Yes	Yes	Pneumonectomy/CCAm type 3	Alive
Case 2	Srinivasan et al., 2012 [27]	No	23 + 4, twin	Yes	Yes	Wedge resection/PPIE	Alive
Case 3	Balegar et al., 2012 [28]	Yes	28	Yes	Yes	Wedge resection/CCAM type 1	Alive
Case 4	Abusalah et al., 2019 [8]	No	28 + 1, twin	Yes	No	−	Alive
Case 5	Chandrasekaran et al., 2021 [29]	Yes	26 + 1	Yes	No	CCAM type 3	Dead
Case 6	Ottomeyer et al., 2023 [9]	No	28	Yes	Yes	Lobectomy/CCAM type 3	Alive
Present case	This study	No	28 + 4	Yes	No	−	Alive

## Data Availability

The original contributions presented in the study are included in the article, further inquiries can be directed to the corresponding author.

## References

[1] Kunisaki S.M. (2021). Narrative review of congenital lung lesions. Transl. Pediatr..

[2] Kotecha S., Barbato A., Bush A., Claus F., Davenport M., Delacourt C., Deprest J., Eber E., Frenckner B., Greenough A. (2012). Antenatal and Postnatal Management of Congenital Cystic Adenomatoid Malformation. Paediatr. Respir. Rev..

[3] Stocker J.T. (2002). Congenital pulmonary airway malformation—A new name for and an expanded classification of congenital cystic adenomatoid malformation of the lung. Histopathology.

[4] Ali M., Tank N., Bawa M. (2023). Antenatally Detected Thoracic Lesions: Prognosis, Management and Outcome. Afr. J. Paediatr. Surg..

[5] Alamo L., Reinberg O., Vial Y., Gudinchet F., Meuli R. (2013). Comparison of foetal US and MRI in the characterisation of congenital lung anomalies. Eur. J. Radiol..

[6] Leblanc C., Baron M., Desselas E., Phan M.H., Rybak A., Thouvenin G., Lauby C., Irtan S. (2017). Congenital pulmonary airway malformations: State-of-the-art review for pediatrician’s use. Eur. J. Pediatr..

[7] Kersten C.M., Hermelijn S.M., Mullassery D., Muthialu N., Cobanoglu N., Gartner S., Bagolan P., Burgos C.M., Sgrò A., Heyman S. (2022). The Management of Asymptomatic Congenital Pulmonary Airway Malformation: Results of a European Delphi Survey. Children.

[8] Abusalah Z.G., Shehab M., Abdalla A., Nakib G.N. (2019). Postnatally presented and spontaneously resolved congenital pulmonary airway malformation in a preterm baby. BMJ Case Rep..

[9] Ottomeyer M., Huddleston C., Berkovich R.M., Brink D.S., Koenig J.M., Sobush K.T. (2023). Early resection of a rare congenital pulmonary airway malformation causing severe progressive respiratory distress in a preterm neonate: A case report and review of the literature. BMC Pediatr..

[10] Fogarty M., Osborn D.A., Askie L., Seidler A.L., Hunter K., Lui K., Simes J., Tarnow-Mordi W. (2018). Delayed vs early umbilical cord clamping for preterm infants: A systematic review and meta-analysis. Am. J. Obstet. Gynecol..

[11] Gornall A.S., Budd J.L.S., Draper E.S., Konje J.C., Kurinczuk J.J. (2003). Congenital cystic adenomatoid malformation: Accuracy of prenatal diagnosis, prevalence and outcome in a general population. Prenat. Diagn..

[12] Ehrenberg-Buchner S., Stapf A.M., Berman D.R., Drongowski R.A., Mychaliska G.B., Treadwell M.C., Kunisaki S.M. (2013). Fetal lung lesions: Can we start to breathe easier?. Am. J. Obstet. Gynecol..

[13] Pederiva F., Rothenberg S.S., Hall N., Ijsselstijn H., Wong K.K.Y., von der Thüsen J., Ciet P., Achiron R., D’adamo A.P., Schnater J.M. (2023). Congenital lung malformations. Nat. Rev. Dis. Prim..

[14] Mehl S.C., Short W.D., Kinley A., Olutoye O.O., Lee T.C., Keswani S.G., King A. (2022). Maternal steroids in high-risk congenital lung malformations. J. Surg. Res..

[15] Baumgarten H.D., Flake A.W. (2019). Fetal Surgery. Pediatr. Clin. N. Am..

[16] Bose S.K., Stratigis J.D., Ahn N., Pogoriler J., Hedrick H.L., Rintoul N.E., Peranteau W.H. (2023). Prenatally diagnosed large lung lesions: Timing of resection and perinatal outcomes. J. Pediatr. Surg..

[17] Montgomery A., Peiffer S., Mehl S., Lee T.C., Keswani S.G., King A. (2024). Management and Outcomes of Patients with High-Risk (Congenital Lung Malformation Volume Ratio ≥ 1.6) Congenital Lung Malformations. J. Surg. Res..

[18] MacGillivray T.E., Harrison M.R., Goldstein R.B., Adzick N.S. (1993). Disappearing fetal lung lesions. J. Pediatr. Surg..

[19] Lima J.S., Camargos P.A.M., Aguiar R.A.L.P., Campos A.S., Aguiar M.J.B. (2014). Pre and perinatal aspects of congenital cystic adenomatoid malformation of the lung. J. Matern. Neonatal Med..

[20] Kunisaki S.M., Ehrenberg-Buchner S., Dillman J.R., Smith E.A., Mychaliska G.B., Treadwell M.C. (2015). Vanishing fetal lung malformations: Prenatal sonographic characteristics and postnatal outcomes. J. Pediatr. Surg..

[21] Meizner I., Rosenak D. (1995). The vanishing fetal intrathoracic mass: Consider an obstructing mucous plug. Ultrasound Obstet. Gynecol..

[22] Crombleholme T.M., Coleman B., Hedrick H., Liechty K., Howell L., Flake A.W., Johnson M., Adzick N. (2002). Cystic adenomatoid malformation volume ratio predicts outcome in prenatally diagnosed cystic adenomatoid malformation of the lung. J. Pediatr. Surg..

[23] Pogoriler J., Swarr D., Kreiger P., Adzick N.S., Peranteau W. (2019). Congenital Cystic Lung Lesions: Redefining the Natural Distribution of Subtypes and Assessing the Risk of Malignancy. Am. J. Surg. Pathol..

[24] Stocker J.T. (2009). Cystic Lung Disease in Infants and Children. Fetal Pediatr. Pathol..

[25] Lezmi G., Hadchouel A., Khen-Dunlop N., Vibhushan S., Benachi A., Delacourt C. (2013). Congenital cystic adenomatoid malformations of the lung: Diagnosis, treatment, pathophysiological hypothesis. Rev. Pneumol. Clin..

[26] Prendergast B., Fernando A.M.R., Mankad P.S. (1998). Congenital cystic adenoid malformation in a pre-term infant: Management considerations. Pediatr. Surg. Int..

[27] Srinivasan R., Ali H., Harigopal S. (2012). Persistent pulmonary interstitial emphysema presenting as solitary lung cyst in a preterm infant. BMJ Case Rep..

[28] Balegar V.K.K., Barr P.A., McCauley J.C., Thomas G. (2013). Selective bronchial intubation in a preterm infant with congenital cystic adenomatoid malformation and pulmonary air leak syndrome. J. Paediatr. Child Health.

[29] Chandrasekaran P., Goergen S., Robinson A., Moghimi A., Malhotra A. (2021). Bilateral congenital pulmonary airway malformation in an extremely preterm infant. BMJ Case Rep..

